# High-Throughput Multiparallel Enteropathogen Detection via Nano-Liter qPCR

**DOI:** 10.3389/fcimb.2020.00351

**Published:** 2020-07-15

**Authors:** Jessica A. Grembi, Koshlan Mayer-Blackwell, Stephen P. Luby, Alfred M. Spormann

**Affiliations:** ^1^Department of Civil and Environmental Engineering, Stanford University, Stanford, CA, United States; ^2^Division of Infectious Diseases and Geographic Medicine, Stanford University, Stanford, CA, United States; ^3^Department of Chemical Engineering, Stanford University, Stanford, CA, United States

**Keywords:** enteropathogen, quantification, qPCR, enteric infection, molecular detection, high-throughput

## Abstract

Quantitative molecular diagnostic methods can effectively detect pathogen-specific nucleic acid sequences, but costs associated with multi-pathogen panels hinder their widespread use in research trials. Nano-liter qPCR (nL-qPCR) is a miniaturized tool for quantification of multiple targets in large numbers of samples based on assay parallelization on a single chip, with potentially significant cost-savings due to rapid throughput and reduced reagent volumes. We evaluated a suite of novel and published assays to detect 17 enteric pathogens using a commercially available nL-qPCR technology. Amplification efficiencies ranged from 88 to 98% (mean 91%) and were reproducible across four operators at two separate facilities. When applied to fecal material, assays were sensitive and selective (99.8% of DNA amplified were genes from the target organism). Due to nanofluidic volumes, detection limits were 1–2 orders of magnitude less sensitive for nL-qPCR than an enteric TaqMan Array Card (TAC). However, higher detection limits do not hinder detection of diarrhea-causing pathogen concentrations. Compared to TAC, nL-qPCR displayed 99% (95% CI 0.98, 0.99) negative percent agreement and 62% (95% CI 0.59, 0.65) overall positive percent agreement for presence of pathogens across diarrheal and non-diarrheal fecal samples. Positive percent agreement was 89% among samples with concentrations above the nL-qPCR detection limits. nL-qPCR assays showed an underestimation bias of 0.34 log_10_ copies/gram of stool [IQR −0.40, −0.28] compared with TAC. With 12 times higher throughput for a sixth of the per-sample cost of the enteric TAC, the nL-qPCR chip is a viable alternative for enteropathogen quantification for studies where other technologies are cost-prohibitive.

## Introduction

Quantitative molecular diagnostic methods, such as quantitative polymerase chain reaction (qPCR), can target nucleic acid gene sequences specific to known microbial pathogens. These methods have provided insights in the study of diarrheal disease beyond what can be gained using microbiological cell culture or immunoassays (van den Berg et al., 2007; Liu et al., 2016b; Platts-Mills et al., 2018) and have been applied successfully in the field of pathogen detection for decades (Wood et al., 1994; Lin et al., 2000; He et al., 2002). Over time, molecular diagnostics were developed from single-gene qPCR assays to multiplex reactions (Soumet et al., 1999; Taniuchi et al., 2011; Mondal et al., 2012) and to multi-assay, multi-sample arrays that can be operated in parallel on a single chip or card (Liu et al., 2013, 2016a; Huang et al., 2016; Wongboot et al., 2018). Specifically in the field of enteric pathogen detection, a TaqMan Array Card (TAC) was developed by Liu et al. (2013, 2016a) and subsequently used in several studies to estimate pathogen-attributable diarrhea burdens (Platts-Mills et al., 2014, 2018; Liu et al., 2016b), as well as the impact of enteric pathogens on child growth (Platts-Mills et al., 2017; Rogawski et al., 2018; Schnee et al., 2018) and vaccine uptake (Grassly et al., 2016; Taniuchi et al., 2016). However, despite advances in the throughput of molecular detection of pathogens, costs associated with broad multi-target molecular assays still pose a barrier to their widespread use in epidemiological research studies. For instance, the per-sample cost of the enteric TAC is $60, not including labor, capital equipment, or DNA extraction reagents (Liu et al., 2013).

Compared with TAC, higher-throughput microfluidic qPCR technologies hold potential to decrease per sample costs of multi-target diagnostics and reduce instrument backlogs for large research studies. In the case of nano-liter (nL) qPCR, precision robotic dispensing permits smaller reaction volumes, increases throughput, and reduces reagent volumes. While nL-qPCR technologies have been previously applied to pathogen detection, early efforts to develop nL-qPCR pathogen chips were limited by factors such as: (i) high limits of detection associated with small reaction volumes (6–33 nL), (ii) insufficient information provided to evaluate quantitative assay validation, and (iii) relatively low sample throughput per chip (12–48 samples; Stedtfeld et al., 2008; Goldfarb et al., 2013; Ishii et al., 2013).

In more recent studies, a commercial nL-qPCR technology (SmartChip™ Real-Time PCR, TakaraBio Inc.) was used to design multi-target diagnostics to detect the presence of antibiotic resistance genes in urban wastewater treatment plant effluent, reclaimed water, and environmental samples (Wang et al., 2014; Karkman et al., 2016; Stedtfeld et al., 2016) and to evaluate a suite of related dehalogenase genes in complex microbial communities (Mayer-Blackwell et al., 2014). This technology uses 100 nL reaction volumes and allows for flexible configuration of a 5,184-well chip that can analyze up to 384 samples (depending on the number of assays included). Using this platform, we developed a nL-qPCR chip targeting 17 enteric pathogens across 96 samples in duplicate. Here, we present a comprehensive evaluation of the technology with laboratory standards as well as fecal samples from children in rural Bangladesh. The nL-qPCR enteropathogen chip permits high-throughput, rapid pathogen detection at significantly lower cost per-sample than other methods.

## Methods

### Assay Design and nL-qPCR Procedures

We selected bacterial, protozoan, and helminthic enteropathogens identified as contributing to diarrheal disease in children across 12 countries (Kotloff et al., 2013; Platts-Mills et al., 2015). We computationally designed primer pairs to target 16 virulence genes using methods described previously (26; and Supplementary Materials). We included an additional 10 published assays (see Table 1) to assess the suitability for inclusion of previously validated assays optimized at similar PCR conditions (Niesters, 2002; Maeda et al., 2003; Verweij et al., 2004; Yu et al., 2005; Wiria et al., 2010; Hoffmann et al., 2013; Liu et al., 2013). The final chip tested 96 samples against 54 assays. The chip was configured to contain 21 pathogen virulence/marker gene targets, three general targets (for bacteria, archaea, and fungi), and four quality control targets. The quality control assays were included to monitor inhibition and efficiency of DNA extraction and qPCR amplification. These included three gene targets from *Mus musculus (ACAA2, B2M, and ESRRA)*, which we have used previously to monitor qPCR amplification inhibition between samples and across chips (Mayer-Blackwell et al., 2014), and a single extrinsic control from phocine herpesvirus (PhHV, *gB*), which is often spiked into DNA lysis buffers prior to sample processing to monitor DNA extraction efficiency (Liu et al., 2016a; Platts-Mills et al., 2018; Rogawski et al., 2018). Each assay was included in duplicate on a single chip (except for two of the four quality control assays, *B2M* and *ESRRA*, which were included once each due to space limitations). Assays were initially evaluated against 490bp synthesized linear DNA strands (Integrated DNA Technologies, Inc., Coralville, IA) for each target gene. Synthetic constructs were designed from reference genes (Table S1) and were used instead of cultured organisms in order to ensure high-precision, equimolar concentrations of each target in the final standard pool. Oligonucleotide primers (Integrated DNA Technologies, Inc., Coralville, IA) at a final concentration of 1 μM were added to a mixture of LightCycler 480 SYBR Green I Master Mix (Roche Applied Sciences, Indianapolis, IN) and the *Mus musculus* control spike-ins (*ESRRA* at 3 x 10^5^ copies/uL, *ACAA2* at 3 x 10^6^ copies/uL, and *B2M* at 3 x 10^7^ copies/uL) and were robotically dispensed onto nL-qPCR chips using TakaraBio's SmartChip™ platform. In a separate plate, samples were added to additional master mix and robotically dispensed onto chips. Duplicate chips were run, using the standard TakaraBio protocol: 95°C for 3 min, then 40 cycles of (95°C for 60 s, 60°C for 70 s). Each chip contained a minimum of two negative (no template) controls for each assay.

**Table 1:** Assays included on the nL-qPCR pathogen chip.

**Organism**	**Gene target**	**Sequence (5^**′**^-3^**′**^)**	**References**
**Pathogenic** ***Escherichia coli***			
Enteroaggregative *E. coli* (EAEC)	*aggR*	F: CAGCGATACATTAAGACGCCT	This study
		R: TCCTTTTGACCAATTCGGACA	
Enterotoxigenic *E. coli* (ETEC)	STh (*estA*)	F: TTCACCTTTCGCTCAGGATG	This study
		R: CCCGGTACAAGCAGGATTAC	
	STp (*estA*)	F: ACTGAATCACTTGACTCTTCAAAAG	This study
		R: ACAACAAAGTTCACAGCAGTAAA	
	LT (*eltA*)	F: CCTGGATTCATCATGCACCA	This study
		R: TCTGGGTCTCCTCATTACAAGT	
Enteropathogenic *E. coli* (EPEC)	*bfpA*	F: GCGAAAGGCTACGGTGTTAA	This study
		R: GCCTCAGCAGGAGTAATAGC	
	*eaeA*	F: GGTCAGATTCAGCATAGCGG	This study
		R: CGCGAGCGGTCACTTTATAA	
Shiga toxin-producing *E. coli* (STEC)	*stx1*	F: ACAGATGGAATCTTCAGTCTCTTC	This study
		R: CTGAATCCCCCTCCATTATGAC	
	*stx2*	F: CTGTTAATGCAATGGCGGC	This study
		R: TGCAGTAACGGTTGCAGATT	
**Other Bacteria**			
*Campylobacter jejuni/coli*	*cdtA*	F: AAAGGATTTGGCGATGCTAGA	This study
		R: CCGCTGTATTGCTCATAGGG	
*Clostridium difficile*	*tcdB*	F: GGTATTACCTAATGCTCCAAATAG	Liu et al., 2016a
		R: TTTGTGCCATCATTTTCTAAGC	
*Clostridium perfringens*	*CPE*	F: GCTGCTGCTACAGAAAGATTAAA	This study
		R: AAGCTTTTGAGTCCAAGGGT	
*Helicobacter pylori*	*ureA*	F: AACTCGTAACCGTGCATACC	This study
		R: TGCCTTCGTTGATAGTGATGT	
*Salmonella enterica*	*invA*	F: TTGACGGTGCGATGAAGTTT	This study
		R: CCACCGAAATACCGCCAATA	
*Shigella* sp./enteroinvasive *E. coli* (EIEC)	*ipaH*	F: GTCAGAAGCCGTGAAGAGAA	This study
		R: TTCAGTACAGCATGCCATGG	
*Vibrio cholerae*	*tcpA*	F: ACACGATAAGAAAACCGGTCA	This study
		R: GCCTTGGTCATATTCTGCGA	
*Yersinia enterocolitica*	*yadA*	F: GCCCAGAAAGATGGAGTAGC	This study
		R: CGTGACTAGAGTGTCCAATGG	
**Protozoa and Helminthes**			
*Cryptosporidium* spp.	18S rRNA	F: GGGTTGTATTTATTAGATAAAGAACCA	Liu et al., 2016a
		R: AGGCCAATACCCTACCGTCT	
*Entamoeba histolytica*	18S rRNA	F: ATTGTCGTGGCATCCTAACTCA	Verweij et al., 2004
		R: GCGGACGGCTCATTATAACA	
*Giardia lamblia*	18S rRNA	F: GACGGCTCAGGACAACGGTT	Verweij et al., 2004
		R: TTGCCAGCGGTGTCCG	
*Ascaris lumbricoides*	ITS1	F: GTAATAGCAGTCGGCGGTTTCTT	Wiria et al., 2010
		R: GCCCAACATGCCACCTATTC	
*Trichuris trichiura*	18S rRNA	F: TTGAAACGACTTGCTCATCAACTT	Liu et al., 2016a
		R: CTGATTCTCCGTTAACCGTTGTC	
**General**			
Total bacteria	16S rRNA	F: GTGSTGCAYGGYTGTCGTCA	Maeda et al., 2003
		R: ACGTCRTCCMCACCTTCCTC	
Total archaea	16S rRNA	F: ATTAGATACCCSBGTAGTCC	Yu et al., 2005
		R: GCCATGCACCWCCTCT	
Total fungi	ITS1	F: CTTGGTCATTTAGAGGAAGTAA	Hoffmann et al., 2013
		R: GCTGCGTTCTTCATCGATGC	
**Quality Control**			
Phocine herpesvirus-1 (PhHV)	*gB*	F: GGGCGAATCACAGATTGAATC	Liu et al., 2013
		R: GCGGTTCCAAACGTACCAA	
*Mus musculus*	*ACAA2*	F: ACAGATACGCCTTGCAGTC	Mayer-Blackwell et al., 2014
		R: CTGTTTGCCTTTCTTCGTCTTC	
	*B2M*	F: GGTCTTTCTGGTGCTTGTCT	Mayer-Blackwell et al., 2014
		R: ACGTAGCAGTTCAGTATGTTCG	
	*ESRRA*	F: CCTGCAAAGCCTTCTTCAAG	Mayer-Blackwell et al., 2014
		R: GTCTCCGCTTGGTGATCTC	

*The chip contains each assay in duplicate, except for two of the Quality Control assays (ACAA2 and ESRRA)*.

### Analytical Performance

Analytical performance was evaluated in accordance with the Minimum Information for Publication of Quantitative Real-Time PCR Experiments (MIQE) guidelines (Bustin et al., 2009). Assay efficiencies were evaluated with a pool of synthetic DNA standards, described above. Standards were 10-fold serially diluted (10–10^6^ copies/reaction). Standard curves were run on a minimum of 15 chips over two instruments at separate facilities (Fremont, CA and East Lansing, MI) and with two different operators at each location. Efficiencies were calculated according to Rutledge and Côté (2003); mean efficiency over all runs is reported along with coefficient of variation. Limit of detection (LOD) was determined with pooled synthetic DNA standards spiked into extracted DNA from 10 fecal samples to a final concentration of 10, 100, and 1,000 copies/reaction; each sample was run in duplicate on two chips. The mean cycle quantification (C_q_) value (i.e., the cycle at which sufficient copies of target DNA have been made to produce a fluorescent signal detectable by the instrument) was calculated for duplicate assays on a single chip, and all results under the C_q_ cutoff of 30 were determined positive. A total of 20 positive samples (10 samples × 2 chips) per target at each concentration were assayed and LOD was defined as the lowest concentration which 95% were positively detected (i.e., where 19 of the 20 were detected).

Inter-assay precision (reproducibility) was assessed across the standard curves used for efficiency calculations measured over 15–20 chips, using different lots of master mix, different batches of oligonucleotide primers, and four different operators at two separate facilities. We report the mean coefficient of variation on calculated copy numbers over all points on the standard curve as well as the range. Intra-assay precision (repeatability) was measured within-chip and between chips. Within-chip precision was evaluated in three samples in which extracted DNA from fecal samples was mixed with positive controls at high (10^5^ copies/reaction) and low (100 copies/reaction) concentrations and assayed 10 times each on a single chip: we report the coefficient of variation of calculated copy number across the 10 replicates. Between-chip precision was evaluated for synthetic DNA standards, for 60 fecal samples into which positive controls were spiked, and for 249 fecal samples collected from a cohort of Bangladeshi children that tested positive for at least one pathogen by TAC. Replicates for each sample were run on two chips and the coefficient of variation of calculated template copies was determined across all four replicates. We report mean coefficient of variation of calculated template copies over all samples, as well as the number of unique samples included in the calculation of the mean.

Sensitivity and specificity were evaluated using DNA standards spiked into extracted DNA from 40 pathogen-free fecal samples. For each pathogen target 10 samples contained the target at low concentration (100 copies/reaction), 10 samples at medium concentration (10x the LOD) and 10 samples at high concentration (100x the LOD); an additional 10 samples had no target. Sensitivity and specificity were determined based on positive or negative detection in these 40 samples. In order to further verify assay specificity, we sequenced qPCR amplicons from 94 sample mixtures which contained a total of 102 Bangladeshi child fecal samples, and 27 positive control samples obtained from other labs at Stanford (see Acknowledgments). The Seq-Ready™ TE MultiSample FLEX protocol, PCR clean-up, and DNA quantification prior to sequencing were done in accordance with TakaraBio's standard procedures, as described previously (Atshemyan et al., 2017; Firtina et al., 2017). The resulting paired-end Illumina MiSeq reads were quality filtered and only sequences that were the expected target gene amplicon length (± 3 bp) were maintained. We verified the intended target (organism and gene) by conducting a nucleotide BLAST search (Altschul et al., 1990) on each unique sequence. We retained the top hit(s), defined as the highest sequence identity with the lowest E value.

### Sample Collection

We used 249 fecal samples from children 10–18 months old in rural Bangladesh to test the performance of nL-qPCR chip against the performance of enteric TAC. Children were enrolled in a randomized controlled trail evaluating the impact of water, sanitation, handwashing, and nutritional interventions on child growth and health (Arnold et al., 2013; Luby et al., 2018; Stewart et al., 2018; Lin et al., 2020); 218 (88%) children did not have diarrheal symptoms in the previous 7 days. Samples were collected by the child's caregiver into a sterile collection container and placed on cold chain within 165 [IQR 79, 791] min, transported to the laboratory and held at −80°C prior to analysis. DNA was extracted according to previously published protocols (Liu et al., 2016a) in the Parasitology lab at the International Centre for Diarrhoeal Disease Research, Bangladesh (icddr,b), including spike-in of 10^6^ copies of PhHV into the lysis buffer, and separated into two aliquots: one aliquot was subjected to TAC analysis at icddr,b and the other was shipped on dry ice to Stanford University. Samples were collected after obtaining written, informed consent from the child's primary caregiver and with approval from human subjects committees at icddr,b (PR-11063), University of California, Berkeley (2011-09-3652), and Stanford University (25863).

### Statistical Analyses

Data analysis was performed in R statistical software, v3.5.2 (R Core Team, 2018) and analysis files are available as Supplementary Materials. Coefficient of variation (the standard deviation of replicates divided by the mean) was used to evaluate precision in accordance with the MIQE guidelines (Bustin et al., 2009). Specifically, coefficient of variation of calculated copy number, and not C_q_ value, is reported per Schmittgen and Livak (2008) and Hellemans et al. (2007). Within-chip, between-chip, and between instrument/operator variances were compared with a pairwise Wilcoxon rank sum test, using the Benjamini-Hochberg procedure to account for multiple comparisons (Benjamini and Hochberg, 1995). Sensitivity and specificity were calculated using the epi.test function from the epiR package (Stevenson et al., 2019). Positive percent agreement and negative percent agreement were calculated in the same manner and are reported with this alternative nomenclature as recommended when no absolute reference standard is used (Food and Drug Administration, 2007). Exact binomial 95% confidence limits on sensitivity and specificity were calculated according to David (1999). Unweighted Cohen's Kappa was calculated using the epi.kappa function with confidence intervals calculated according to Rothman (2002). Bias in calculated log_10_ copy numbers per gram of stool (corrected for extraction and PCR efficiency by normalizing to the positive control PhHV spike-in) was evaluated according to Martin Bland and Altman (1986) using the blandr::blandr.statistics function to estimate bias (Datta, 2017); 95% confidence intervals determined per Bland and Altman (2002).

## Results

### Analytical Performance

The mean efficiency for each assay, based on the evaluation of standard curves run on 15–20 chips, ranged from 88 to 98% (mean 91%) with a coefficient of variation of 6.3% [IQR 5.3, 7.3] (Table 2). The linearity over all assays on all chips was 0.990 [IQR 0.987, 0.992] and detection limits were between 10 and 100 copies/100 nL reaction, which corresponds to 8 × 10^5^-8 × 10^6^ copies/g of stool (Table 2). Within-chip repeatability was assessed in 10 replicates on a single chip: synthetic DNA in high (10^5^ copies/reaction) and low (10^2^ copies/reaction) concentrations was spiked into DNA extracted from fecal samples. The high concentration displayed a coefficient of variation in calculated copy number of 15% [IQR 8–25]; the low concentration had variability of 27% [IQR 18–36] (Figure S1).

**Table 2:** Analytical performance of the nL-qPCR pathogen chip.

**Organism (gene target)**	**Efficiency % (CV1^a^)**	**LOD^b^ copies/g (copies/reaction)**	**Reproducibilty CV above, at LOD^c^**	**Repeatability CV (n)^d^**	**Sensitivity**	**Specificity**
**Pathogenic** ***Escherichia coli***						
EAEC (*aggR*)	95 (0.16)	8e+05 (10)	26, 115	19 (75)	100	93
ST-ETEC (STh)	90 (0.07)	8e+06 (100)	22, 51	44 (8)	100	100
ST-ETEC (STp)	92 (0.08)	8e+06 (100)	28, 39	28 (13)	100	100
LT-ETEC (*eltA*)	91 (0.08)	8e+06 (100)	24, 49	18 (25)	100	100
EPEC (*bfpA*)	89 (0.05)	8e+06 (100)	26, 43	32 (13)	100	100
EPEC (*eaeA*)	90 (0.05)	8e+05 (10)	23, 55	33 (59)	100	93
STEC (*stx1*)	89 (0.08)	8e+06 (100)	18, 47	46 (6)	100	100
STEC (*stx2*)	92 (0.08)	8e+06 (100)	22, 57	62 (16)	100	100
**Other Bacteria**						
*Campylobacter jejuni/coli* (*cdtA*)	92 (0.07)	8e+06 (100)	32, 52	29 (35)	100	100
*Clostridium difficile* (*tcdB*)	90 (0.05)	8e+06 (100)	20, 34	25 (8)	100	100
*Clostridium perfringens* (CPE)	88 (0.06)	8e+05 (10)	22, 52	24 (2)	98	100
*Helicobacter pylori* (*ureA*)	91 (0.07)	8e+06 (100)	28, 92	42 (2)	100	100
*Salmonella enterica* (*invA*)	91 (0.07)	8e+05 (10)	21, 53	68 (3)	100	100
*Shigella*/EIEC (*ipaH*)	90 (0.07)	8e+06 (100)	20, 29	25 (15)	100	100
*Vibrio cholerae* (*tcpA*)	89 (0.06)	8e+06 (100)	20, 44	41 (1)	100	100
*Yersinia enterocolitica* (*yadA*)	92 (0.07)	8e+06 (100)	26, 61	64 (2)	100	100
**Protozoa and Helminthes**						
Cryptosporidium (18S rRNA)	88 (0.07)	8e+06 (100)	36, 103	76 (4)	100	100
Entamoeba histolytica (18S rRNA)	90 (0.1)	8e+06 (100)	28, 46	54 (2)	100	100
Giardia (18S rRNA)	90 (0.07)	8e+06 (100)	21, 31	33 (17)	100	90
Ascaris lumbricoides (ITS1)	89 (0.05)	8e+05 (10)	20, 53	16 (2)	100	100
Trichuris trichiura (18S rRNA)	91 (0.07)	8e+05 (10)	17, 58	28 (2)	100	100
**General and Quality Control**						
Total Archaea (16S rRNA)	90 (0.06)	8e+06 (100)	44, 75	70 (2)	100	93
Total Bacteria (16S rRNA)	98 (0.09)	NA	31, 319	NA	NA	NA
Total Fungi (ITS1)	91 (0.1)	8e+06 (100)	43, 54	58 (34)	100	87
PhHV (*gB*)	92 (0.07)	8e+06 (100)	22, 66	52 (92)	100	100

a *Coefficient of variation (CV) of efficiency calculated across 15–20 chips*.

b *minimum number of gene copies per gram of stool (minimum number of gene copies per 100 nL reaction)*.

c *CV on calculated copy number for all points along the standard curve measured over 15-20 chips, shown separately for concentrations above the LOD and at the LOD*.

d *CV in calculated copy number across four replicates measured in n positive samples, mean CV is reported*.

**Figure 1:**
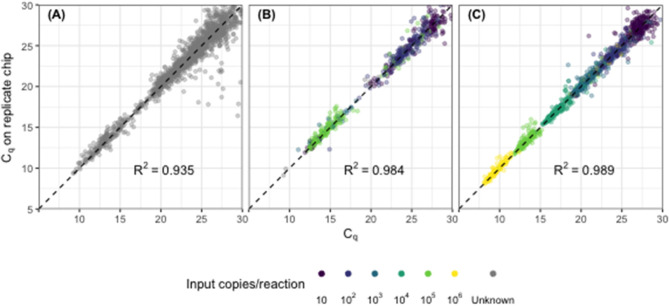
Assay precision across replicate chips for **(A)** synthetic DNA standards across a 6-fold dilution series, **(B)** synthetic DNA standards spiked into child fecal samples (*n* = 60), and **(C)** child fecal samples (*n* = 249). Each point represents the replicated results for a single sample-assay reaction run in a specific location on the chip. Points shown with color indicate results from amplified DNA standards with defined input copy number (10–10^6^); gray points indicate results from fecal samples with unknown input copy number, absent synthetic standards.

C_q_ values across replicate chips were highly repeatable for synthetic DNA standards (*R*^2^ = 0.989, Figure 1A), for synthetic DNA in a complex stool DNA matrix (*R*^2^ = 0.984, Figure 1B) and for DNA extracted from fecal samples collected from children in Bangladesh (*R*^2^ = 0.935, Figure 1C). Fecal samples displayed a median difference in C_q_ values of 0.39 [IQR 0.15–0.81] (Figure 1C) across all assays, which corresponds to a coefficient of variation on calculated gene copy number of 28% [IQR 16–50] (Table 2, Repeatability). The highest variability was again seen at the lowest concentrations (Figure S2).

Assays were reproducible across two instruments and four operators, with the same inverse relationship noted between variance and concentration (Table S3). At concentrations one or more orders of magnitude above the detection limit, coefficient of variation on calculated copy number ranged from 17 to 44% (Table 2). Coefficient of variation at the limit of detection ranged from 29 to 115% for pathogen virulence and marker genes, the highest of which was analogous to 17 ± 20 copies detected. Variance for the total bacterial (16S rRNA) assay at the detection limit (10 copies/reaction) was highest at 319%. Between-chip variance was similar to variance across two instruments and four operators (*p* = 0.99) but both were significantly higher than within-chip variance (*p* < 0.0001, pairwise Wilcoxon rank sum test). Coefficients of variation of the magnitudes observed are not biologically relevant when analyzing pathogen quantities on the log_10_ scale, as is the normal procedure.

Analytical sensitivity ranged from 98 to 100% and specificity from 90 to 100% (Table 2) among 40 samples containing combinations of synthetic nucleic acid spiked into DNA extracted from 10 different individuals and assayed in duplicate. To further ensure the specificity of the assays, we sequenced amplicons from 96 fecal samples collected from children in Bangladesh that tested positive for at least one pathogen target. We obtained 1.7M (26,747 unique) sequences with 330 [IQR 142, 1,171] unique sequences per assay. Amplicon sequencing showed that the assays were specific. The intended gene target was correctly identified in the top hit(s) (defined as highest identity and lowest *E*-value) for 99.8% of unique sequences. Most (99.7%) of the BLASTn searches returned a database top hit with ≥97% sequence identity. The *Ascaris lumbricoides* assay had highest number of off-target hits: 7/130 of the unique sequences were identified as the same target gene in a closely related species, *Ascaris ovis*.

### Clinical Performance

For 249 Bangladeshi child fecal samples, overall percent agreement between nL-qPCR and TAC was 91% for the >4,400 reactions and negative percent agreement was 99% [95% CI 98, 99; Cohen's Kappa = 0.69 (95% CI 0.66–0.72)]. Positive percent agreement was highly dependent on concentration of the target gene. At concentrations above nL-qPCR detection limits (>10^7^ copies/g stool) positive percent agreement was 89%; this dropped to 61% for concentrations near the nL-qPCR detection limits (10^5^-10^7^ copies/ g stool) and fell to 5% for concentrations below 10^5^ copies/g stool (Table S4). In instances where both methods detected the presence of target genes, nL-qPCR assays displayed a median underestimation bias of −0.34 log_10_ copies [IQR −0.40, −0.28] (see Table 3 and Figure S3 for individual assay statistics).

**Table 3:** Bland-Altman bias estimates by assay on calculated log_10_ copy number per gram of stool for nL-qPCR compared to TAC.

**Pathogen (gene target1^a^)**	**n1^b^**	**Bias (95% CI)**
EAEC (*aggR*)	92	–1.1 (–1.2, –1)
ST-ETEC (STh)	8	0.1 (0, 0.2)
ST-ETEC (STp)	24	−0.6 (−0.9, −0.3)
LT-ETEC (*eltA*)	37	−0.8 (−1, −0.6)
EPEC (*bfpA*)	17	−0.7 (−0.8, −0.5)
EPEC (*eaeA*)	74	−0.1 (−0.3, 0)
STEC (*stx1*)	5	0.4 (0, 0.8)
STEC (*stx2*)	9	0.7 (−0.1, 1.6)
Campylobacter jejuni/coli (cdtA)	47	0.1 (−0.1, 0.2)
Clostridium difficile (tcdB)	8	−1 (−1.7, −0.3)
Salmonella enterica (invA)	1	−0.1 (NaN, NaN)
*Shigella* sp./EIEC (*ipaH*)	17	−1.1 (−1.2, −1.0)
Cryptosporidium (18S)	6	−1.1 (−2.4, 0.2)
Giardia (18S)	16	−1.6 (−2.1, −1.1)

*The table shows the 14 organisms targeted on both platforms for which there was at least 1 sample with concordant detection. EAEC, enteroaggregative Escherichia coli; EIEC, enteroinvasive E. coli; EPEC, enteropathogenic E. coli; LT-ETEC, enterotoxigenic E. coli with heat-labile toxin; NaN, non a number (due to n = 1); STEC, shiga toxin-producing E. coli; ST-ETEC, enterotoxigenic E. coli with heat-stable toxin*.

a *Gene target from nL-qPCR chip (not always the same gene target as the TAC)*.

b *Number of the 249 samples for which this organism was detected via both TAC and nL-qPCR*.

**Figure 2:**
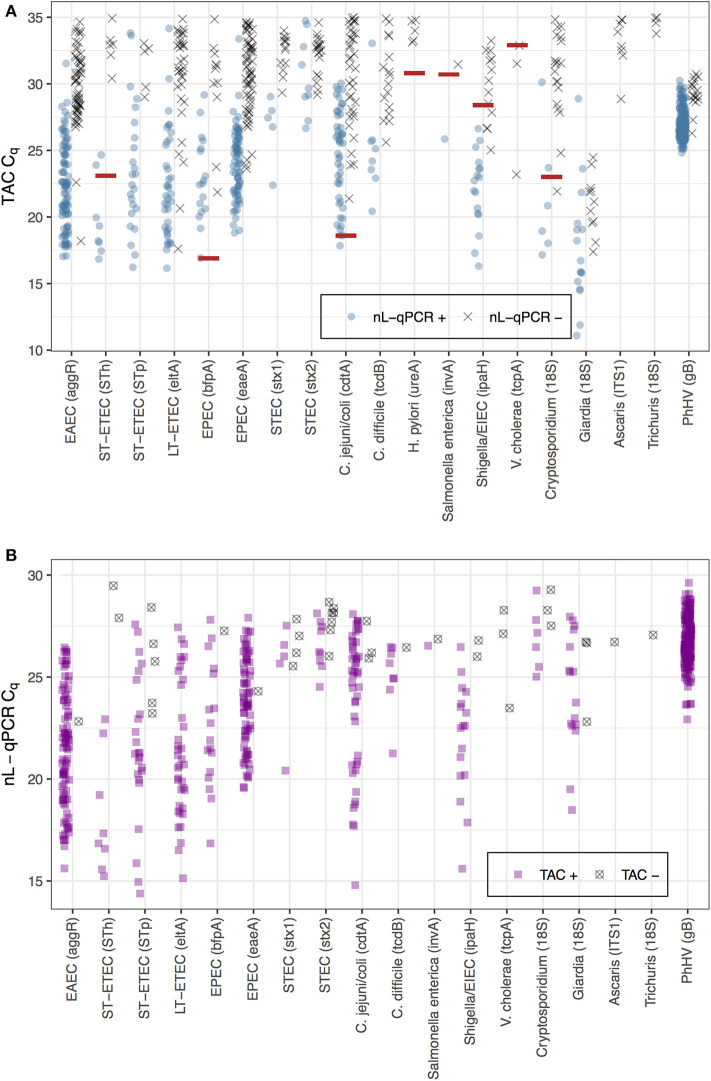
Comparison of nL-qPCR and TAC assays across 249 fecal samples. Samples detected by **(A)** TAC or **(B)** nL-qPCR are shown with their respective C_q_ values. Blue (top) and purple (bottom) points represent positive detections found in both nL-qPCR and TAC tests. Gray points (top) represent targets undetected by nL-qPCR but detected by TAC, and vise versa (bottom). Red lines represent pathogen TAC etiologic cutoff C_q_ values (etiologic cutoffs have not been established for nL-qPCR assays).

The average TAC C_q_ value was 30.9 [IQR 28.6, 33.0] for reactions detected by TAC but not by nL-qPCR, which was just above the maximum detectable cycle (C_q_ = 30) for nL-qPCR (Figure 2A, black points). The higher detection limits for nL-qPCR assays did not interfere with detection of diarrhea-causing pathogen concentrations, with the exception of the *V. cholerae* assay which had an etiologic cutoff that was below the nL-qPCR detection limit. The etiologic cutoff (shown as red lines in Figure 2A) indicates the TAC C_q_ value below which children were highly likely to have diarrhea, i.e., the value at which the odds ratio for diarrhea cases compared to controls was >2 (Liu et al., 2016b; Platts-Mills et al., 2018). nL-qPCR assays detected all but eight of the 40 reactions in which TAC assays detected a sample below the etiologic C_q_ cutoff value (4 for *Shigella*/EIEC, 3 for *V. cholerae*, and 1 for *Cryptosporidium*), and typically detected samples well above the cutoff for most assays (Figure 2A). Reactions positive by nL-qPCR but not TAC were also at low concentrations (Figure 2B) and could have been the result of less stringent amplification without the use of probe-based dyes with nL-qPCR.

Contamination may cause false-positive qPCR results, and can occur due to cross-contamination between samples or as a result of free ambient DNA in the laboratory environment. Sample cross-contamination occurred rarely with nL-qPCR; amplification of pathogen virulence or marker genes in no-template controls occurred in <3% of the 4,288 no-template control sample reactions. Moreover, these amplifications resulted in calculated copy numbers near or below the established limit of detection (35 copies; IQR 28–42 copies). Ambient laboratory contamination was detected more frequently and was highly dependent on operator. Amplification of bacterial 16S rRNA occurred in 46% of no-template controls, but always at concentrations near the detection limit [11 (IQR 7, 25) copies]. Cross-contamination, although possible, occurs rarely and only in low concentration, thereby indicating a low likelihood of false positive results.

## Discussion

The nL-qPCR chip evaluated here provides satisfactory analytical performance for simultaneous analysis of 96 samples against a suite of 17 enteric pathogens for a cost of < $10/sample. The high-throughput nature of the nL-qPCR chip is particularly advantageous when large numbers of samples need to be processed in a timely manner, such as in population-based research studies and field trials. Above certain thresholds, we found analytical performance to be comparable to an enteric TAC widely used for investigations of diarrheal disease in diverse global populations, for at least a quarter of the per-sample cost ($60; Liu et al., 2013). The cost of TAC can be greater if not purchased in bulk (Table S2). nL-qPCR is not likely to detect pathogens shed at concentrations below the detection limits (8 × 10^5^–8 × 10^6^ copies/g stool).

The primary difference in performance we observed was that a majority of the nL-qPCR assays had detection limits 1–2 orders of magnitude higher than TAC. The reduction in sensitivity is caused primarily by the fact that the nL-qPCR reaction volumes are 0.0125 μL compared to 0.2–0.4 μL for TAC; (10, personal communication with Liu, 2019). Among 249 fecal samples from Bangladeshi children, most nL-qPCR assays displayed a modest underestimation bias (i.e., returned a lower estimated number of copies per gram of stool) compared to the enteric TAC. However, these differences do not appear to be limitations in terms of ability to distinguish pathogen loads relevant for diarrheal disease for pathogens with established etiological cutoffs, with the potential exception of *Vibrio cholerae*. Importantly, the TAC and nL-qPCR assays for *V*. *cholerae* target different virulence genes: hemolysin (*hlyA*) for TAC and toxin-coregulated pilus (*tcpA*) for nL-qPCR. The etiologic cutoffs were established for *hlyA*, which is commonly detected in environmental *V. cholerae* strains that lack both the *tcpA* and cholera toxin genes (Hasan et al., 2013). Thus, discordant detection between the technologies might not represent differences in performance, but rather differential presence of these virulence genes within *V. cholerae* strains. Given that studies have shown low concentrations of *V. cholerae hlyA* gene are observed in feces coincident with diarrheal symptoms in children (Liu et al., 2016b; Platts-Mills et al., 2018), this might be a superior gene target for *V*. *cholerae* in pathogen panels. Additional studies should verify the optimal gene target for diarrhea-causing *V. cholerae* species. Not including the *V. cholerae* assay, the nL-qPCR chip was able to detect 32 of 37 (86%) samples with targets below TAC etiologic cutoffs, despite the lower sensitivity due to the chip's higher limits of detection. Future work could address the nL-qPCR assays that failed to identify *Shigella*/EIEC (4 samples) and *Cryptosporidium* (1 sample) in samples where TAC identified quantities below the established etiologic cutoffs.

In studies where quantitation is required at lower concentrations than were achieved in this study, pre-amplification can be performed as described by Ishii et al. (2013). In addition, pre-printing primers directly onto chips, similar to the TAC spotting procedure, can reduce detection limits by nearly 50%. However, a major advantage of the nL-qPCR SmartChip™ is the flexibility of the platform. Therefore, if a research team does opt to pre-print primers onto chips, we suggest also maintaining a stock of unprinted chips. The current configuration of the chip was designed with large-scale epidemiology studies in mind, thus increased throughput was prioritized over the inclusion of a higher number of assays. Researchers could increase throughput even further if focusing on a smaller set of targets, which would permit more samples per chip and reduce per-sample costs. In large-scale studies, replicating analysis for questionable samples is often necessary (e.g., when replicates give discordant results). Unprinted chips allow for an operator to run a limited suite of sample/assay pairs that need to be reanalyzed: for example, 384 samples with questionable results in the initial run from a large study could be analyzed against a minimal suite of 12 assays on a specially designed chip at the end of the study. This facilitates the resolution of discordant results and minimizes missing values in the final dataset to maximize statistical power in the analysis stage.

Unprinted nL-qPCR chips also allow end-users, with appropriate assay validation, to substitute assays in the set reported here. Our evaluation included 10 pre-published assays that operate at similar PCR conditions. We found they performed well in nL format, suggesting that end users have flexibility in re-designing the chip. We further show that seven primer pairs previously validated using TaqMan with probe-based dyes had excellent specificity among 96 fecal samples when utilized with SYBR Green intercalating dye instead. These results suggest the additional reagent costs associated with probes may not be necessary in some circumstances to achieve high specificity, which is consistent with other reports of equal or superior specificity with SYBR Green compared to TaqMan chemistry (Maeda et al., 2003; Peng et al., 2018).

Quantifying nucleic acid targets for large numbers of samples is expensive, regardless of the platform used and the tradeoff between technical replicates and biological replicates is often debated in large studies. Although technical replicates are generally encouraged (the rule of thumb is triplicates for qPCR), these are often sacrificed in the face of limited budgets to ensure greater statistical power afforded in the analysis with independent biological replicates (Kitchen et al., 2010; Taylor et al., 2010). Technical replicates are important to facilitate identification of outlier or spurious results, particularly on chip- or card-style platforms, and increase the likelihood of detection near the detection limit where analytical precision is the lowest (Smyth et al., 2005; Yuan and Irizarry, 2006; Liu et al., 2013). The nL-qPCR pathogen chip is configured to provide duplicate results for the 21 pathogen-specific virulence and marker genes. This was deliberate as it is impossible to determine *a priori* if a sample will be near the detection limit, particularly in the case of fecal samples where the presence of PCR inhibitors is likely (Monteiro et al., 1997; Wilson, 1997). Early versions of the enteric TAC included replicates (Liu et al., 2013), but those have been replaced by additional pathogen targets in latter versions currently in use for large-scale studies (Liu et al., 2016b; Platts-Mills et al., 2018). Due to the flexibility in configuration of the nL-qPCR, up to 13 additional pathogen targets could be added without sacrificing duplicate assays, and throughput would still be 8–9 times higher and cost 50% less than the enteric TAC. It is our hope that the lower per-sample cost and built-in technical replicates may facilitate best practices under budgetary constraints.

The nL-qPCR platform has important limitations. First, nL-qPCR does not appear to be well-suited for absolute quantification of total bacteria due to the fact that general bacterial contamination (measured by quantification of the 16S rRNA gene with broad specificity primers) was detected near the detection limit in almost half of the no-template control samples. Due to the open chip technology, there is higher likelihood for contamination if not used in a controlled laboratory with minimal ambient contamination and operators meticulous in their practice of sterile technique. To ensure potential low-concentration contamination is identified, we strongly recommend incorporation of replicates, as included on the nL-qPCR chip evaluated here, when using this technology or more stringent C_q_ filtering (e.g., C_q_ 29 or earlier). Secondly, the use of robotic liquid-handling instruments and specialized thermocyclers require a high capital investment, which might make the technology inaccessible to some institutions. Capital costs of these instruments are comparable to those required for the TAC, and availability of the instruments at shared user facilities might facilitate cost-sharing across labs. Another limitation of molecular methods in general is the inability to determine presence of viable organisms. However, model-derived quantitative cutoffs could be established for nL-qPCR based on the odds ratio of having clinical diarrheal symptoms at a specific C_q_ value, as has already been done for TAC (Platts-Mills et al., 2015; Liu et al., 2016b). Additionally, the importance of asymptomatic enteropathogen detection via molecular methods is increasing due the associations observed with childhood stunting and vaccine efficacy (Grassly et al., 2016; Taniuchi et al., 2016; Platts-Mills et al., 2017; Rogawski et al., 2018). Finally, the current nL-qPCR chip configuration does not include viral enteric pathogen targets. The primary aim for this study was to validate the nL-qPCR technology for bacterial and parasitic targets, and future iterations of the chip including viral targets could be combined with a reverse-transcriptase protocol for the study of RNA as well as DNA viruses.

In conclusion, we found the nL-qPCR pathogen chip to be an acceptable alternative for population-based field trials interested in enteric pathogen outcomes; the savings in both cost and time will be amplified at scale.

## Data Availability Statement

The original contributions presented in the study are publicly available. This data can be found here: https://www.ncbi.nlm.nih.gov/bioproject/; PRJNA593072.

## Ethics Statement

The studies involving human participants were reviewed and approved by human subjects committees at icddr,b (PR-11063), University of California, Berkeley (2011-09-3652), and Stanford University (25863). Written informed consent to participate in this study was provided by the participants' legal guardian/next of kin.

## Author Contributions

JG and AS conceived of the study. JG and KM-B planned the study and designed primers. SL and JG obtained fecal samples from Bangladesh. JG conducted lab work, analyzed data and wrote the manuscript. All authors contributed to manuscript revision, read, and approved the final manuscript.

## Conflict of Interest

The authors declare that the research was conducted in the absence of any commercial or financial relationships that could be construed as a potential conflict of interest.
