# Supplementary material for: High-Throughput Multiparallel Enteropathogen Detection via Nano-Liter qPCR
Source: Front Cell Infect Microbiol. 2020 Jul 15;10:351. doi: 10.3389/fcimb.2020.00351 (PMC7381150; doi:10.3389/fcimb.2020.00351)
Supplement: Supplementary file 2 [file Data_Sheet_1.docx]

Supplementary Material

## Supplementary Methods

*Assay design and pre-screening*

We computationally designed and screened 175,000 candidate primer pairs to target 16 virulence genes using methods described previously (Mayer-Blackwell et al., 2014). Briefly, amino acid sequences corresponding to all non-redundant members of each target gene’s protein family (Pfam v 27.0) were clustered based on percent pairwise identity using BLASTp all-vs-all search (Altschul et al., 1990). We downloaded corresponding nucleotide sequences from NCBI, and DNA oligonucleotide primers were designed to target conserved DNA-level sequence motifs in sequence clusters containing the target virulence protein for each pathogen (Table S1). A custom Python script directed the software primer3 (Untergasser et al., 2012) to develop thousands of candidate primer pairs for each target gene, which were then screened *in silico* against other non-target clusters within the same protein family via the freely available EMBOSS program fuzznuc (Rice et al., 2000). The scripts used for this primer design workflow are available, along with a docker image containing the software to run the scripts, on GitHub (<https://github.com/jgrembi/nL-qPCR_PathogenChip>). Up to eight assays per target gene were selected for laboratory screening. During laboratory screening, assays that reproducibly displayed fluorescence in the negative control (PCR grade water) prior to cycle 28, failed to amplify standards at 100 copies per well, or had PCR efficiencies less than 85% were immediately excluded from further consideration. Final assays were selected based on optimal performance characteristics, as described in the main text.

## Supplementary Tables

**Table S1**. Synthetic constructs used as standards for nL-qPCR. 490bp synthetic standards were used as opposed to clinical isolates or ATCC strains to allow for a precise copy numbers to be added to a single standard pool containing identical quantities of each target. Each standard was derived from a specific GenBank accession. For assays developed under this project, the standard was derived from a different accession than that used to design primers. For each accession, a 490bp region of the gene of interest that contained the PCR-targeted region was selected such that the target locations for primer attachment were not within 50bp of the end of the 490bp sequence. Due to synthesis requirements imposed by the manufacturer (Integrated DNA Technologies), regions of unacceptable complexity (e.g., GC content) that were outside the PCR targeted region were modified in a few cases, as noted.

*(See separate excel file: TableS1_SyntheticStandards.xlsx)*

**Table S2**. Cost comparison of consumable supplies for nL-qPCR enteric pathogen chip and enteric TaqMan Array Card (TAC). Nonconsumable costs include a specialized thermocycler instrument (QuantStudio for TAC; SmartChip Cycler for nl-qPCR) and additional instruments for sample handling (centrifuge with TAC-supported bucket adapters for TAC; SmartChip MultiSample Nano Dispenser robotic fluid handling system for nL-qPCR). Cost estimates obtained from Stanford University purchasing system in August 2019.

| **Characteristic** | **nL-qPCR pathogen chip** | **Enteric TAC** |
| --- | --- | --- |
| No. reaction wells | 5184 | 384 |
| No. samples (per chip or card) | 96 | 8 |
| Cost (per chip or card) | $475 | $600 |
| Mastermix ^a^ cost (per sample) | $1.30 | $78 |
| Primer^b^ cost (per sample) | $0.43 | NA (spotted on TAC) |
| Consumables-tips, tubes, 96-well plates (per sample) | $3.00 | $1.50 |
| **TOTAL (per sample)** | **$9.68** | **$155** |
| ^a^ AgPath-ID One-Step RT-PCR Kit (1000 reactions; $1600) for TAC; Roche LightCycler 480 SYBR Green I (103680 reactions; $2500) for nL-qPCR  ^b^ IDT primer plate with mixed forward and reverse primers at 50uM (good for >6 nL-qPCR chips; $250) | | |

**Table S3.** Coefficient of variation in target gene copy number across 15-20 chips that were run on two instruments at separate facilities, by two operators at each facility. Synthetic DNA standards were evaluated over a dilution series from 10 to 10^6^ copies/reaction. Coefficient of variation for 10 copies/reaction is not shown when the assay limit of detection was determined to be higher than 10 copies/reaction.

|  | **Target copy number** | | | | | |
| --- | --- | --- | --- | --- | --- | --- |
| **Organism (gene target)** | **10** | **10^2^** | **10^3^** | **10^4^** | **10^5^** | **10^6^** |
| **Pathogenic *Escherichia coli*** | | | | | | |
| EAEC (*aggR*) | 115.3 | 0.9 | 26.1 | 25.0 | 16.3 | 61.8 |
| ST-ETEC (STh) |  | 51.1 | 45.0 | 22.7 | 18.4 | 20.5 |
| ST-ETEC (STp) |  | 39.3 | 38.8 | 25.9 | 22.7 | 29.5 |
| LT-ETEC (*eltA*) |  | 48.7 | 39.4 | 26.8 | 19.8 | 22.2 |
| EPEC (*bfpA*) |  | 43.3 | 54.2 | 31.8 | 17.2 | 19.8 |
| EPEC (*eaeA*) | 55.3 | 35.5 | 73.2 | 23.0 | 15.4 | 20.2 |
| STEC (*stx1*) |  | 47.2 | 18.2 | 21.0 | 13.8 | 16.8 |
| STEC (*stx2*) |  | 57.3 | 21.8 | 23.5 | 14.6 | 22.8 |
| **Other Bacteria** | | | | | | |
| *Campylobacter jejuni/coli* (*cdtA*) |  | 51.6 | 44.4 | 29.7 | 21.8 | 33.3 |
| *Clostridium difficile* (*tcdB*) |  | 34.0 | 19.2 | 27.4 | 15.8 | 21.9 |
| *Clostridium perfringens* (CPE) | 52.1 | 45.8 | 22.4 | 22.8 | 16.0 | 21.7 |
| *Helicobacter pylori* (*ureA*) |  | 92.4 | 25.8 | 35.3 | 13.8 | 31.1 |
| *Salmonella enterica* (*invA*) | 53.1 | 31.3 | 23.1 | 20.6 | 12.2 | 18.1 |
| *Shigella*/EIEC (*ipaH*) |  | 29.4 | 19.4 | 20.4 | 13.8 | 21.2 |
| *Vibrio cholerae* (*tcpA*) |  | 44.0 | 22.3 | 25.4 | 15.4 | 18.8 |
| *Yersinia enterocolitica* (*yadA*) |  | 60.8 | 101.0 | 29.0 | 16.6 | 21.6 |
| **Protozoa & Helminthes** | | | | | | |
| Cryptosporidium (18S rRNA) |  | 102.9 | 112.3 | 40.1 | 31.5 | 19.7 |
| Entamoeba histolytica (18S rRNA) |  | 45.9 | 25.6 | 28.6 | 35.6 | 23.7 |
| Giardia (18S rRNA) |  | 31.5 | 19.5 | 21.7 | 12.9 | 27.6 |
| Ascaris lumbricoides (ITS1) | 53.2 | 30.6 | 19.1 | 21.6 | 14.7 | 20.2 |
| Trichuris trichiura (18S rRNA) | 57.7 | 31.8 | 17.2 | 21.4 | 12.5 | 17.2 |
| **General & Quality Control** | | | | | | |
| Total Archaea (16S rRNA) |  | 74.6 | 44.7 | 49.5 | 31.4 | 42.8 |
| Total Bacteria (16S rRNA) | 318.9 | 78.2 | 91.0 | 29.7 | 16.7 | 30.5 |
| Total Fungi (ITS1) |  | 54.5 | 40.1 | 53.5 | 44.8 | 40.5 |
| PhHV (*gB*) |  | 66.1 | 25.8 | 22.9 | 15.4 | 21.8 |

# Abbreviations: EAEC, enteroaggregative *Escherichia coli*; EIEC, enteroinvasive *E. coli*; EPEC, enteropathogenic *E. coli*; LT-ETEC, enterotoxigenic *E. coli* with heat-labile toxin; STEC, shiga toxin-producing *E. coli*; ST-ETEC, enterotoxigenic *E. coli* with heat-stable toxin

## Table S4. Clinical performance comparison between nL-qPCR and TAC on 249 child fecal samples. Values in parenthesis are 95% confidence intervals. Performance was best above nL-qPCR detection limits (>10^7^ copies/g stool).

|  |  | **All samples** | | | | | **>10^7^ copies/g^b^** | | | **10^5^-10^7^ copies/g^b^** | | | **<10^5^ copies/g^b^** | | |
| --- | --- | --- | --- | --- | --- | --- | --- | --- | --- | --- | --- | --- | --- | --- | --- |
| **Organism (gene target)** | **n^a^** | **n TAC^+^** | **Overall percent agreement** | **Cohen's kappa** | **Negative percent agreement** | **Positive percent agreement** | **n TAC^+^** | **Positive percent agreement** | **n TAC^+^** | | **Positive percent agreement** | **n TAC^+^** | | **Positive percent agreement** |  |
| **Pathogenic *E. coli*** | | | | | | | | | | | | | | |  |
| EAEC (*aggR*) | 223 | 151 | 73.1 | 0.49  (0.38, 0.61) | 98.6  (92.5, 100) | 60.9  (52.7, 68.8) | 88 | 96.6  (90.4, 99.3) | 56 | | 12.5  (5.2, 24.1) | 7 | | 0  (0, 41) |  |
| ST-ETEC (STh) | 227 | 14 | 96.5 | 0.65  (0.52, 0.78) | 99.1  (96.6, 99.9) | 57.1  (28.9, 82.3) | 7 | 100  (59, 100) | 2 | | 50  (1.3, 98.7) | 5 | | 0  (0, 52.2) |  |
| ST-ETEC (STp) | 207 | 29 | 95.2 | 0.8  (0.66, 0.94) | 97.2  (93.6, 99.1) | 82.8  (64.2, 94.2) | 19 | 100  (82.4, 100) | 10 | | 50  (18.7, 81.3) | 0 | | NA |  |
| LT-ETEC (*eltA*) | 213 | 70 | 84.5 | 0.6  (0.48, 0.72) | 100  (97.5, 100) | 52.9  (40.6, 64.9) | 37 | 86.5  (71.2, 95.5) | 25 | | 16  (4.5, 36.1) | 8 | | 12.5  (0.3, 52.7) |  |
| EPEC (*bfpA*) | 235 | 28 | 94.9 | 0.71  (0.59, 0.84) | 99.5  (97.3, 100) | 60.7  (40.6, 78.5) | 17 | 88.2  (63.6, 98.5) | 10 | | 20  (2.5, 55.6) | 1 | | 0  (0, 97.5) |  |
| EPEC (*eaeA*) | 223 | 135 | 72.2 | 0.48  (0.37, 0.59) | 98.9  (93.8, 100) | 54.8  (46, 63.4) | 41 | 92.7  (80.1, 98.5) | 63 | | 55.6  (42.5, 68.1) | 31 | | 3.2  (0.1, 16.7) |  |
| STEC (*stx1*) | 227 | 18 | 92.5 | 0.34  (0.21, 0.46) | 98.1  (95.2, 99.5) | 27.8  (9.7, 53.5) | 1 | 100  (2.5, 100) | 5 | | 80  (28.4, 99.5) | 12 | | 0  (0, 26.5) |  |
| STEC (*stx2*) | 214 | 28 | 87.9 | 0.35  (0.22, 0.47) | 96.2  (92.4, 98.5) | 32.1  (15.9, 52.4) | 0 | NA | 16 | | 37.5  (15.2, 64.6) | 12 | | 25  (5.5, 57.2) |  |
| **Other Bacteria** | | | | | | | | | | | | | | |  |
| *Campylobacter* *jejuni*/*coli* (*cdtA*) | 215 | 83 | 81.9 | 0.59  (0.46, 0.71) | 97.7  (93.5, 99.5) | 56.6  (45.3, 67.5) | 23 | 87  (66.4, 97.2) | 41 | | 65.9  (49.4, 79.9) | 19 | | 0  (0, 17.6) |  |
| *Clostridium difficile* (*tcdB*) | 235 | 24 | 92.8 | 0.45  (0.34, 0.57) | 99.5  (97.4, 100) | 33.3  (15.6, 55.3) | 8 | 87.5  (47.3, 99.7) | 12 | | 8.3  (0.2, 38.5) | 4 | | 0  (0, 60.2) |  |
| *Helicobacter pylori* (*ureA*) | 233 | 5 | 97.9 | 0  (0, 0) | 100  (98.4, 100) | 0  (0, 52.2) | 0 | NA | 0 | | NA | 5 | | 0  (0, 52.2) |  |
| *Salmonella enterica* (*invA*) | 238 | 2 | 99.2 | 0.5  (0.37, 0.62) | 99.6  (97.7, 100) | 50  (1.3, 98.7) | 0 | NA | 1 | | 100  (2.5, 100) | 1 | | 0  (0, 97.5) |  |
| *Shigella*/EIEC (*ipaH*) | 232 | 30 | 93.5 | 0.66  (0.54, 0.78) | 99  (96.5, 99.9) | 56.7  (37.4, 74.5) | 20 | 85  (62.1, 96.8) | 10 | | 0  (0, 30.8) | 0 | | NA |  |
| *Vibrio* *cholerae* (*tcpA*) | 238 | 3 | 97.5 | -0.01  (-0.14, 0.11) | 98.7  (96.3, 99.7) | 0  (0, 70.8) | 1 | 0  (0, 97.5) | 0 | | NA | 2 | | 0  (0, 84.2) |  |
| **Protozoa & Helminthes** | | | | | | | | | | | | | | |  |
| *Cryptosporidium* (18S rRNA) | 228 | 25 | 90.4 | 0.31  (0.2, 0.43) | 98.5  (95.7, 99.7) | 24  (9.4, 45.1) | 6 | 83.3  (35.9, 99.6) | 8 | | 12.5  (0.3, 52.7) | 11 | | 0  (0, 28.5) |  |
| *Entamoeba* *histolytica*  (18S rRNA) | 220 | 0 | 96.8 | 0  (0, 0) | 96.8  (93.6, 98.7) | NA | 0 | NA | 0 | | NA | 0 | | NA |  |
| *Giardia* (18S rRNA) | 162 | 27 | 91.4 | 0.65  (0.5, 0.8) | 97.8 (93.6, 99.5) | 59.3  (38.8, 77.6) | 23 | 60.9  (38.5, 80.3) | 4 | | 50  (6.8, 93.2) | 0 | | NA |  |
| *Ascaris* *lumbricoides* (ITS1) | 220 | 8 | 95.9 | -0.01  (-0.09, 0.07) | 99.5  (97.4, 100) | 0  (0, 36.9) | 0 | NA | 1 | | 0  (0, 97.5) | 7 | | 0  (0, 41) |  |
| *Trichuris* *trichiura* (18S rRNA) | 239 | 4 | 97.9 | -0.01  (-0.11, 0.09) | 99.6  (97.7, 100) | 0  (0, 60.2) | 0 | NA | 1 | | 0  (0, 97.5) | 3 | | 0  (0, 70.8) |  |
| Abbreviations: EAEC, enteroaggregative *Escherichia coli*; EIEC, enteroinvasive *E. coli*; EPEC, enteropathogenic *E. coli*; LT-ETEC, enterotoxigenic *E. coli* with heat-labile toxin; NA, not applicable; STEC, shiga toxin-producing *E. coli*; ST-ETEC, enterotoxigenic *E. coli* with heat-stable toxin ^a^ Number of samples with valid results for both TAC and nL-qPCR  ^b^ Subset restricted to samples with specified copy number per gram of stool, calculated via TAC C_q_ value and standard curve | | | | | | | | | | | | | | | |

## Supplementary Figures

**Figure S1.** Within-chip intra-assay precision among samples containing a mixture of synthetic DNA standards and extracted DNA from 3 separate fecal samples. Each sample was assayed 10 times on a single chip. Synthetic DNA was added at either (a) 100 or (b) 10^5^ copies/reaction. Points represent the mean value across the 10 replicates and error bars show the standard deviation. The total bacteria results are not shown as fecal samples contained an unknown quantity of bacterial 16S rRNA which confounds the interpretation.

**Figure S2.** Calculated copy numbers have the highest variation at the lowest concentrations. Boxplots show median value and inner quartile ranges, points represent the result for a single sample-assay reaction run in quadruplicate (twice each on two chips), colors indicate if the assay targeted the general bacterial 16S rRNA gene or a specific pathogen virulence/marker gene. *** p < 0.001, ** p < 0.01 as determined by Wilcoxon rank sum test.

**Figure S3.** Bland-Altman plots for assays with at least 10 detections in fecal samples in both nL-qPCR and TAC. The blue shaded area represents the mean bias and its 95% confidence interval. The upper (green) and lower (red) limits of agreement and their corresponding 95% confidence intervals are also shown.

**References**

Altschul, S. F., Gish, W., Miller, W., Myers, E. W., and Lipman, D. J. (1990). Basic local alignment search tool. *J. Mol. Biol.* 215, 403–410. doi:10.1016/S0022-2836(05)80360-2.

Mayer-Blackwell, K., Azizian, M. F., Machak, C., Vitale, E., Carpani, G., de Ferra, F., et al. (2014). Nanoliter qPCR Platform for Highly Parallel, Quantitative Assessment of Reductive Dehalogenase Genes and Populations of Dehalogenating Microorganisms in Complex Environments. *Environ. Sci. Technol.* 48, 9659–9667. doi:10.1021/es500918w.

Rice, P., Longden, L., and Bleasby, A. (2000). EMBOSS: The European Molecular Biology Open Software Suite. *Trends Genet.* 16, 276–277. doi:10.1016/S0168-9525(00)02024-2.

Untergasser, A., Cutcutache, I., Koressaar, T., Ye, J., Faircloth, B. C., Remm, M., et al. (2012). Primer3-new capabilities and interfaces. *Nucleic Acids Res.* 40, e115. doi:10.1093/nar/gks596.
